# Checkpoint Inhibitors Modulate Plasticity of Innate Lymphoid Cells in Peripheral Blood of Patients With Hepatocellular Carcinoma

**DOI:** 10.3389/fimmu.2022.849958

**Published:** 2022-06-27

**Authors:** Bernd Heinrich, Benjamin Ruf, Varun Subramanyam, Yuta Myojin, Chunwei W. Lai, Amanda J. Craig, Jianyang Fu, Changqing Xie, Alexander Kroemer, Tim F. Greten, Firouzeh Korangy

**Affiliations:** ^1^ Thoracic and GI Malignancies Branch, Center for Cancer Research, National Cancer Institute, National Institutes of Health, Bethesda, MD, United States; ^2^ Department of Gastroenterology, Hepatology and Endocrinology, Hannover Medical School, Hannover, Germany; ^3^ Liver Diseases Branch, National Institute of Diabetes and Digestive and Kidney Disease, National Institutes of Health, Bethesda, MD, United States; ^4^ Laboratory of Human Carcinogenesis, Center for Cancer Research, National Cancer Institute, Bethesda, MD, United States; ^5^ MedStar Georgetown Transplant Institute, MedStar Georgetown University Hospital and the Center for Translational Transplant Medicine, Georgetown University Medical Center, Washington, DC, United States; ^6^ National Cancer Institute, Center for Cancer Research (NCI CCR) Liver Cancer Program, National Institutes of Health, Bethesda, MD, United States

**Keywords:** checkpoint inhibitors, anti-PD-1, anti-CTLA-4, innate lymphoid cells, NK-cells, hepatocellular carcinoma, single-cell RNA-sequencing, PBMC

## Abstract

Innate lymphoid cells (ILC) are a heterogeneous and plastic population of cells of the innate immune system. Their role in cancer and specifically in hepatocellular carcinoma is unraveling. The presence of ILCs in peripheral blood of HCC patients has not been explored yet. Their role and function in response to checkpoint inhibitor therapy have also not been explored. Here, we characterized ILCs in PBMC of HCC patients at baseline and after treatment with immune checkpoint inhibitors (ICI) by flow cytometry and single-cell sequencing. Characterization of ILC subsets in PBMCs of HCC patients showed a significant increase in ILC1 and a decrease in ILC3 frequencies. Single-cell RNA-sequencing identified a subgroup of NK-like ILCs which expressed cytotoxicity markers as well as NKp80/*KLRF1*. This *KLRF1^high^* NK-like population showed low abundance in patients with HCC and was enhanced after combined anti-CTLA-4 and anti-PD-1immunotherapy. Trajectory analysis placed this population in between ILC1 and ILC3 cells. The transcriptomic signature of *KLRF1^high^* NK-like ILCs was associated with better progression-free survival in large HCC cohorts. This study shows a previously unknown effect of ICI on the composition and plasticity of ILCS in peripheral blood. Thus, ILCs from PBMC can be used to study changes in the innate immune system under immunotherapy.

## Introduction

Innate lymphoid cells (ILCs), a heterogeneous population of the innate immune system, have been found to play an important role in controlling immune responses (1). ILCs have been studied in the context of various disease settings such as asthma, inflammatory bowel disease, or arthritis (2). Their essential role in cancer has begun to emerge with the need to understand their function in anti-tumor immunity (3, 4). ILCs are more abundant in tissue and only represent a small subset within mononuclear cells in blood. They lack antigen-specific receptors enabling them to provide immediate responses to changes in their environment by secreting cytokines and mediating subsequent adaptive immune responses. Helper ILCs, considered to be functionally comparable to helper T cells are defined as lineage negative (lin^-^) CD127^+^CD161^+^ lymphocytes (5) and can be further divided into ILC1s, ILC2s, and ILC3s (6). ILC1s are not a homogeneous population, and the lack of a specific marker makes their analysis more complex. ILC1s mainly secrete IFNγ and rely on the transcription factor Tbet. ILC2s express the transcription factor GATA-3 which is essential for their differentiation and secrete IL-4, IL-5, IL-9, IL-13 upon activation. ILC3s depend on the transcription factor RORgt and secrete cytokines like IL-17 and IL-22. They can be further divided based on the expression of natural cytotoxicity receptor (NCR), NKp44 into NKp44^+^ and NKp44^-^ subpopulations (1).

Hepatocellular carcinoma (HCC) is an inflammation-triggered cancer (7). Several components of the immune system are involved in development, progression, and treatment of HCC which ultimately influence the prognosis. We recently performed a comprehensive characterization of ILCs in tumor tissue from resections or liver transplants of HCC patients, which revealed a cytokine gradient-driven plasticity of hepatic ILCs in the tumor microenvironment (4).

Current treatment options for HCC include immune checkpoint inhibitors (ICIs) such as the PD-1 inhibitors pembrolizumab (8) and nivolumab (9), PD-L1 inhibitor atezolizumab also in combination with anti-VEGF (10) as well as anti-CTLA-4 antibody, tremelimumab. ILCs have not been studied extensively in the context of immune checkpoint inhibitor therapy of HCC.

In this study, we profiled ILCs from peripheral blood mononuclear cells (PBMCs) from HCC patients and studied the effect of anti-CTLA-4 antibody (tremelimumab) as well as anti-PD-L1 antibody (durvalumab) on these cells. We performed flow cytometry and single-cell sequencing (scRNA-seq) analysis to study ILC subgroups in PBMCs and compared the distribution of ILCs in HCC patients to healthy controls and patients with chronic hepatitis B (HBV) or hepatitis C (HCV) without liver cancer. Finally, we studied the composition of ILC subsets in response to ICI therapy in HCC patients who received tremelimumab and durvalumab therapy. Flow cytometry and transcriptomic profiling revealed a previously unknown cytotoxic NKp80^+^/KLRF1^high^ NK-like ILC population, which was reduced in frequency in PBMCs of HCC patients at baseline. However, upon ICI therapy, NKp80^+^/*KLRF1^high^* NK-like ILC subset was detected at levels similar to healthy donors. Transcriptomic profiling as well as trajectory analysis placed this *KLRF1^high^* NK-like ILC population between ILC1 and ILC3 cells, indicating an intermediate phenotype. We also identified the transcriptomic signature of *KLRF1^high^* NK-like ILCs in liver tissue of HCC patients and this signature was associated with better progression-free survival (PFS) in HCC cohorts. Thus, we present a previously unknown biological effect of ICIs on the composition and plasticity of ILCs in PBMCs. We show that a cytotoxic NKp80^+^/*KLRF1^high^* NK-like ILC subgroup is associated with better survival and is revived upon immunotherapy.

## Materials and Methods

### PBMC Isolation

Peripheral blood was collected from a total of 54 HCC patients. The patients were enrolled in one of two clinical trials at the National Institutes of Health (NIH) evaluating the combination of anti-CTLA4 (tremelimumab) and anti-PD-L1 (durvalumab) or anti-CTLA4 (tremelimumab) alone (NCT02821754 and NCT01853618). Blood was diluted 1:1 with PBS and transferred into Greiner Leucosep Tube (Lonza) that had been loaded with Lymphocyte separation medium (Lonza). Density gradient centrifugation was performed with centrifugation at 2200 RPM for 25 min at room temperature without breaks. Plasma in the supernatant was discarded and PBMCs were recovered from the middle layer by carefully pipetting. Cells were washed with PBS once by centrifugation at 400g for 5 min at 4°C. RBC lysis was applied followed by two washing steps before cell counting. Cells were split and used for immediate flow cytometry analysis, flow sorting and sequencing, or were frozen for future experiments.

### Flow Cytometry Analysis and Cell Sorting

Immune cells were resuspended in staining buffer (BD Biosciences, Franklin Lakes, NJ, USA). Live/dead staining with APC-Cy7 (BD Bioscience) was performed before FC-blocking using human FC blocking reagent (BD Bioscience). Cells were further stained for surface markers as shown in **Table 1**. After incubation for 30 min at 4°C, cells were washed twice in staining buffer. For intracellular staining of cytokines, cells were incubated for 4h at 37°C and 5% CO_2_ using BD Leukocyte Activation Cocktail, with BD GolgiPlug™ per protocol in a 96-well plate. Cells were permeabilized and fixed with the BD fixation/permeabilization kit (BD Bioscience) according to the manufacturer’s instructions. For staining of transcription factors, BD transcription factor buffer set was used (BD Bioscience) for fixation and permeabilization. Cells were further stained for intracellular cytokines or transcription factors. After incubation for 30 min at 4°C, cells were washed twice with staining buffer.

**Table 1:** Antibodies for flow cytometry.

Live/Dead APC-Cy7	ThermoFisher	
Live/Dead Zombie UV	Biolegend	
Fc-block	BD	
CD45 AF700	BD	Clone HI30
CD3 PacBlue (BV421)	BD	Clone UCHT1
CD94 PacBlue (BV421)	BD	Clone HP-3D9
CD34 PacBlue (BV421)	BD	Clone 581
CD123 PacBlue (BV421)	BD	Clone 7G3,
CD1a PabBlue (BV421)	BD	Clone HI149
TCRαβ PacBlue (BV421)	BioLegend	Clone IP26
TCRγδ PacBlue (BV421)	BD	Clone B1
CD14 PacBlue (BV421)	BD	Clone M5E2
CD19 PacBlue (BV421)	BioLegend	Clone HIB19
CD16 PacBlue (BV421)	BD	Clone 3G8
BDCA-2 (CD303) PacBlue (BV421)	BD	Clone V24-785
FcεRIα PacBlue (BV421)	BioLegend	Clone AER-37
CD127 (IL-7α) PE	BD	Clone hIL-7R-M21
CD117 (c-KIT) PE-Cy5	BD	Clone YB5.B8
CD161 (KLRB1)	BD	Clone DX12
CRTH2 (CD294) AF647 (APC)	BD	Clone BM16
NKp44 PE-Cy7	BioLegend	Clone P44-8
CD56 BV605	BioLegend	Clone HCD56
CTLA-4 (CD152) BV786	BD	Clone BNI3
PD1 (CD279) BV605	BD	Clone EH12.1
PD-L1 (CD274) BV650	BD	Clone MIH1
NKp80 APC	BioLegend	Clone 5D12
IFNg BV510	BD	Clone B27
Perforin APC-fire	BioLegend	B-D48
GZMK AF594	BioLegend	GM26E7

Fluorescence was measured using a Cytoflex Flow Cytometer (Beckmann Coulter). For compensation single color staining and for correct gating setting FMOs (fluorescence minus one) were performed. For regular analysis cells were analyzed on Beckman Coulter Cytoflex LX flow cytometer.

Flow cytometry sorting was used to enrich the helper ILC population for sequencing. Cells were stained for CD45 and lineage markers (CD1a, CD34, CD3, TCRαβ, TCRγδ, CD14, CD19, CD16, CD94, CD123, BDCA2, FCeR1a), CD127 and CD161 and then immediately sorted on a fluorescence activated cell sorter type BD Influx or FACSAria. The lineage negative, CD127 positive, CD161 positive population contained the helper ILC enriched population. Again, immediate preparation was crucial for yield and viability of the delicate cell population. Therefore, sorted cells were instantly transferred for cell capture and library preparation.

For Flow cytometry data analysis and illustration FlowJo 10.7.1 (BD Biosciences) was used.

### Statistical Analysis

Quantitative analysis was performed using GraphPad Prism (version 8.42). Means were compared by using Student’s t-test or one-way ANOVA for hypothesis testing to compare individual or corresponding groups (Tukey’s multiple comparison test). Mann–Whitney U or Kruskall-Wallis test (Dunn’s multiple comparison test) were applied if data sets failed the Pearson omnibus normality test (or Shapiro-Wilk normality test if N was too small) (alpha = 0.05). Error bars reflect standard error of the mean SEM. Statistics are reflected if not otherwise declared as non-significant (ns)=p>0.05, *=p<0.05, **=p<0.01, ***=p<0.001, ****p<0.0001.

### Single Cell Capture and cDNA Library Preparation

Single cell gene expression and protein expression analysis was done using BD Rhapsody Single cell analysis platform following manufacturer’s suggested protocol. Briefly, BD cartridge was primed with air, and washing buffers. Freshly isolated and flow sort enriched ILC populations of 1000-10,000 single cells were resuspended in sample buffer (BD Bioscience) before loading onto microwells of the BD Rhapsody cartridges. Cartridge and cells were incubated for 15 min, and cells were scanned in the cartridge. Cell capture beads were loaded onto the cartridge and incubated with the sample following the protocol. Two washing steps were performed before another imaging step. Next, cell lysis buffer was added to the samples and incubated for 2 min. After incubation on the large magnet, reverse transcription was performed (25 min). A targeted panel of primers was used for library preparation. Libraries were sequenced on Illumina NextSeq550.

### Preparation of Single Cell Suspensions From Liver Tissue

Fresh tissue from a total of 11 HCC patients undergoing liver surgery was collected in MACS tissue storage solution (Miltenyi Biotec) (4). Immediate tissue preparation was a crucial factor for yield and viability of isolated cells, therefore, single cell preparation was started directly after tissue collection and finished in an interval of less than 8 hours. Tissue weight was measured, and the digestion process was adapted accordingly. Samples were minced with a sterile blade and incubated with 2ml collagenase IV (1mg/ml, STEMCELL technologies) and 150 µl DNAse I (1mg/ml, STEMCELL Technologies) in a total volume of 10ml RPMI 1640 (Gibco) without FCS per 1g tissue for 90 min at 37°C in a rocking shaker. Cells were spun down at 1400 RPM for 5 min and washed once with RPMI plus FCS before filtering through a 100 µm filter. Cells were washed twice, and a density gradient centrifugation using 30% and 70% Percoll solution was performed to separate the immune cells from the remaining liver tissue. Cells were resuspended in 30% Percoll (GE Healthcare) and layered onto 70% Percoll solution. Centrifugation was performed at 2400 RPM, for 20 min at room temperature without brake. Immune cells were recovered from the middle layer and washed with RPMI once. RBC lysis was performed before cells were counted. Cells were used for immediate flow cytometry analysis, flow sorting and sequencing, or were frozen for future experiments.

### scRNA-Seq Data Processing for Targeted Transcriptomic Data

Targeted capture libraries were prepared using BD Rhapsody Single-cell Analysis System with the targeted human immune response panel of 399 genes (BD Biosciences, https://www.bd.com/documents/specifications/genomics/GMX_BD-Rhapsody-immune-response-human-panel_SP_EN.pdf) adding six additional targets (CCR6 PolyA, CCR6 Reference End, CD19, CLEC4C, KLRD1, NCR2) according to manufacturer’s recommendations ((BD Biosciences). Libraries were pooled and sequenced on NextSeq 550 using pair-end runs (R1:76bps, R2:76bps). The demultiplex of sequencing run was performed using Illumina bcl2fastq version2.17. One mismatch in sample barcodes was allowed to tolerate sequencing error or PCR errors. The average sequencing yield was about 133 million raw reads per sample and these reads were stored in FASTQ format.

The FASTQ-formatted files were processed using the standard Rhapsody analysis pipeline version 1.3 on Seven Bridges (https://www.sevenbridges.com). Mate pairs in which either read was too short or too low in sequencing quality were first removed (if Read1 <66bps or Read2 < 64bps or base call quality score is less than Q20 in either mate). In addition, reads with low complexity such as strings of identical bases and tandem repeats were removed. After filtering, the average total reads per sample was about 125 million reads for scRNA-seq samples. Cell barcodes and unique molecular identifiers (UMIs) were extracted from R1 reads. R2 reads were mapped to reference sequences (Immune Amplicons reference for mRNA-seq) using Bowtie2. The percentages of uniquely aligned reads were between 70% - 90% for each sample. A mate pair was retained if and only both reads survived the above filtering and both reads mapped uniquely.

Finally, all valid R1 and R2 reads were combined and annotated to the respective molecules by applying both recursive substation error correction (RSEC) for collapsing molecules with one base difference in UMIs as well as distribution-based error correction (DBEC) algorithms to remove PCR errors in the UMI sequences from BD Rhapsody analysis pipeline. The mean DBEC sequencing depth was 416x and sequencing saturation was about 88% for scRNA-seq.

Putative cell identification was done using a basic implementation of second derivative analysis to find cut-off point with additional refinement steps in the BD Rhapsody analysis pipeline to remove false positive cells and recover false negative cells. For scRNA-seq samples from PBMCs, the putative cell counts were between 128 – 2477. The mean reads per sample after DBEC correction were about 154,000 reads/cell, and the mean molecule per cells was about 170 molecules/cell. For scRNA-seq samples from liver tissue, the putative cell counts were between 290 – 3037. The mean reads per sample after DBEC correction were about 64,500 reads/cell, and the mean molecule per cells was about 1281 molecules/cell.

The final gene expression matrices contain DBEC-adjusted molecule counts was generated in a CSV format.

### Analysis of Single-Cell RNA Sequencing Data Using Partek Flow

Single-cell RNA sequencing data was imported into Partek Flow in form of filtered DBEC files after pre-processing as described above. Gene specific analysis was performed using Partek^®^ Flow^®^ software, v10.0. Single cell QA and QC was performed filtering for a total read of 1500 counts and no filter for detected genes due to the limited number of genes based on the targeted transcript panel. Noise reduction was implemented by filtering cells with genes expressed in a value of 0.0 in 99.9% of cells. Normalization was performed by log transformation of cpm. Data from remaining B-cell and antigen presenting cells in the population were eliminated from further downstream analysis using immunoglobulin transcripts and B-cell markers CD19 and CD20 or CD14 for antigen presenting cells and monocytes. Clustering was repeated after filtering of contaminating cells. Unbiased graph-based clustering was performed using Louvain algorithm with a resolution of 1 and default settings in Partek flow. UMAP dimensional reduction was performed using default settings. Differential gene expression was done by Partek Flow internal algorithm GSA with false-discovery rate using the Benjamini-Hochberg correction. Trajectory analysis was preformed after clustering using default settings based on Monocle V2 algorithm. Differentially expressed genes were compared using a threshold of a p-value of 0.05 and fold change larger than two. Volcano plots were created using R-studio 1.2. with R version 3.5.1 and the ggplot2 package version 3.3.3.

### Pathway Analysis

Pathway analyses were done using gprofiler on the webpage https://biit.cs.ut.ee/gprofiler/gost. Gene lists of differentially expressed genes using a threshold of p-value <0.05 and fold change larger than two were implemented in gprofiler and pathway analysis using human KEGG database was performed using the recommended tailor-made g:SCS algorithm for multiple testing correction.

### TCGA Survival Analysis

Survival analysis using the TCGA liver cancer cohort was performed using Kaplan Meier-plotter web-based online tool (11). Patients were split at median and no other filters have been applied. Mean expression values of genes were used for recurrence-free survival or PFS analysis. For analysis of patients with HCC, patients pre-treated with sorafenib were chosen for PFS analysis.

## Results

### ILC Composition in Patients With HCC Is Altered

**Figure 1:**
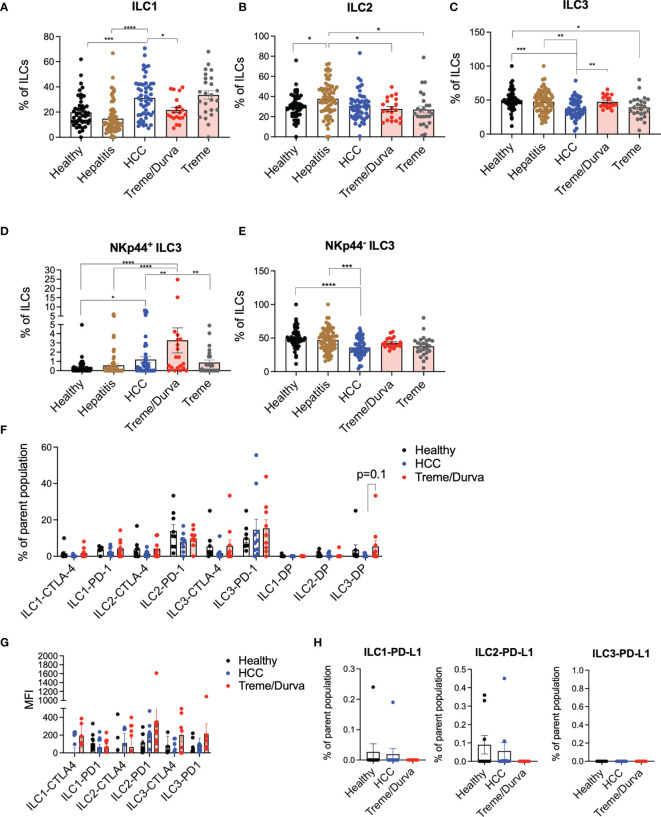
**(A–C)** Flow cytometry (FC) data analysis presenting ILC1, ILC2, and ILC3 frequencies of CD45^+^lin^-^CD127^+^CD161^+^ ILCs. Lin is defined as CD1a, CD34, CD3, TCRαβ, TCRγδ, CD14, CD19, CD16, CD94, CD123, BDCA2, FCeR1a. ILC1s are defined as CD45^+^lin^-^CD127^+^CD161^+^CRTH2^-^c-KIT^-^; ILC2s are defined as CD45^+^lin^-^CD127^+^CD161^+^CRTH2^+^c-KIT^+/-^; ILC3s are defined as CD45^+^lin^-^CD127^+^CD161^+^CRTH2^-^c-KIT^+.^ Cohorts include healthy donors (n = 57), patients with hepatitis (n = 70), patients with HCC before immunotherapy (n = 54), and patients after receiving up to four cycles of their immunotherapy checkpoint inhibitor (ICI) regimen of either combination of tremelimumab and durvalumab (treme/durva, n = 20) or tremlimumab only (treme, n = 26). **(A)** Bar plots showing ILC1 frequency in individual cohorts. **(B)** Bar plots showing ILC2 frequency in individual cohorts. **(C)** Bar plots showing ILC3 frequency in individual cohorts. **(D)** Bar plots showing frequency of NKp44^+^ ILC3s of ILC3s. **(E)** Bar plots showing frequency of NKp44^-^ ILC3s of ILC3s. **(F)** Bar plots showing frequency of ILCs expressing CTLA-4 or PD-1 or CTLA-4/PD-1 double positive (DP) cells by subgroup and separated by cohorts. **(G)** Bar plots showing median fluorescence intensity of CTLA-4 and PD-1 on ILC subgroups separated by cohorts. **(H)** Bar plots showing frequency of ILCs expressing PD-L1 by subgroup and separated by cohorts. Significance: *=p < 0.05, **=p < 0.01, ***=p < 0.001, ****p < 0.0001.

We studied ILCs in peripheral blood from patients with HCC, tumor-free patients with chronic HBV or HCV infection, and healthy controls (**Figures S1A, B**). Lin negative CD161 positive cells were stained for c-KIT, CRTH2, and NKp44 to identify ILC1 (defined as c-KIT^-^, CRTH2^-^), ILC2 (defined as CRTH^+^, c-KIT^+/-^), and ILC3 (defined as CRTH2^-^ and c-KIT^+^) subsets (Gating strategy **Figure S1C**). Amongst the ILC subsets, ILC1 cells showed the highest frequency in HCC patients compared to healthy controls and hepatitis patients (**Figure 1A**; 31.3% vs. 19.8%, p=0.0001, vs. 14.7%, p=<0.00001). The frequency of ILC2s was not significantly changed in HCC patients as compared to healthy donors but it was increased in patients with hepatitis (**Figure 1B**). ILC3 frequency was decreased in HCC patients as compared to healthy controls and hepatitis patients (**Figure 1C** **;** 37.9% vs. 50.15%, p=0.0005, vs. 47.6%, p=0.0041). A more in-depth analysis of ILC3 subsets based on NKp44 expression (12) revealed that the NKp44^+^ ILC3 population was significantly increased in HCC patients as compared to healthy controls (**Figure 1D** **;** 1.2% vs. 0.3%, p=0.017) and NKp44^-^ ILC3s were reduced (**Figure 1E**; 35.9% vs 49.6%, p=<0.0001).

### Tremelimumab Plus Durvalumab Treatment Modulates the Frequency of ILC1 and ILC3 Similar to Levels Seen in Healthy Controls

All HCC samples were obtained from patients enrolled in two different clinical trials evaluating the effect of anti-CTLA4 (Tremelimumab; NCT02821754) or anti-CTLA4 plus anti-PD-L1 treatment (Durvalumab; NCT01853618) (13–15) which opened the possibility to study possible immune checkpoint inhibitor related changes in ILCs (**Figure S1A**). Patient information can be found in **Supplementary Figures S1D–F**. Frequencies of ILCs in peripheral blood of HCC patients from baseline (C1) were compared with samples obtained from patients treated with tremelimumab and durvalumab (treme/durva; up to cycle 4) or tremelimumab treatment alone (up to cycle 4). Flow cytometry analysis revealed that upon combined treme/durva treatment, there was a significant decrease in the frequency of ILC1s (31.3% to 21.6%, p=0.029) and an increase in ILC3 frequency (37.8% to 47.4%, p=0.0063) as compared to baseline (**Figures 1A, C**). Patients treated with ICI showed reduced ILC2 frequencies compared to patients with hepatitis, with no significant changes detected when compared to baseline (**Figure 1B**). To identify which checkpoint inhibitor might mediate the observed effect, we also tested PBMCs from a different trial testing tremelimumab single agent. Tremelimumab alone had no significant effects on ILC1, ILC2, or ILC3 frequencies in peripheral blood when compared to ILC composition in HCC patients before immunotherapy treatment (**Figures 1A–C**).

Next, we analyzed PD-1, CTLA-4, and PD-L1 expression on ILCs. Approximately 15% and 20% of all ILC2s and ILC3 expressed PD-1 (**Figure 1F**) with ILC2 and ILC3 showing the highest median fluorescence intensity of CTLA-4 and PD-1 upon immunotherapy (**Figure 1G**). PD-L1 expression was low on all three ILC subgroups (**Figure 1H**).

In summary, our data indicate that the frequency of ILCs differs in HCC patients as compared to healthy donors or patients with hepatitis and that the combination of tremelimumab and durvalumab treatment changes the ILC frequencies back to its composition in healthy donors. There was a trend toward increased expression of checkpoints PD-1 and CTLA-4 specifically on ILC3s under combined checkpoint inhibitor therapy, indicating that checkpoint inhibitors can mediate ILC phenotype.

### Single-Cell RNA Sequencing Reveals a Cytotoxic NK-Like ILC Population With ILC1-ILC3 Intermediate Phenotype

We performed single-cell RNA sequencing to further characterize ILCs in PBMCs in our patient cohort. ILCs are a rare population of cells which are mainly tissue resident and which require enrichment for characterization of their heterogeneity and subsets. We used the BD Rhapsody platform with a targeted panel of immune system-related transcripts as previously described (4, 16). This offers the opportunity to study sparse cell populations in detail and identify biologically relevant subclusters which cannot be identified by low parameter analyses. ILCs (CD45^+^lin^-^CD127^+^CD161^+^ cells) were enriched and sorted prior to scRNA-seq as we have recently described (4). A total of 4118 cells derived from nine patients were sequenced (**Figures S1A, C**).

**Figure 2:**
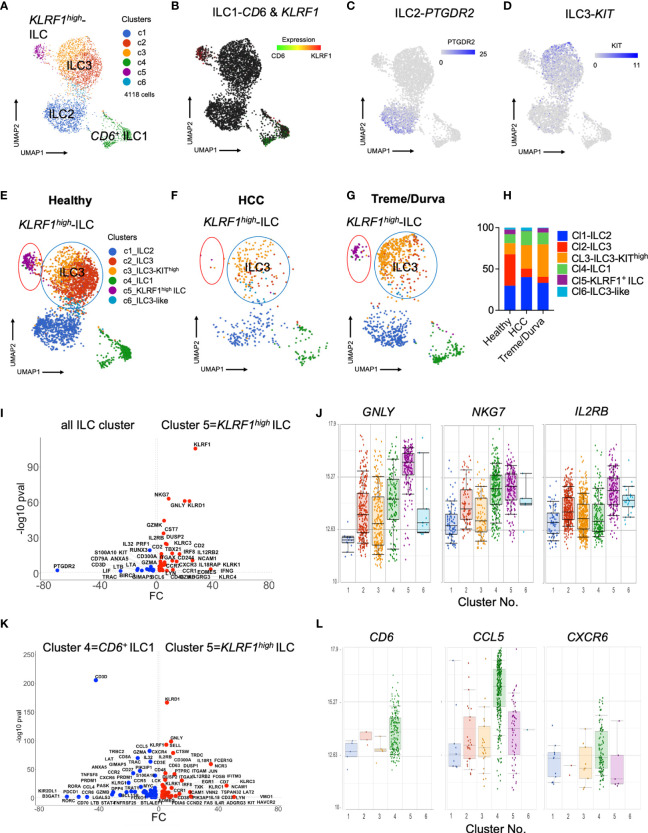
**(A)** UMAP plot of scRNA-seq data of lin^-^CD127^+^CD161^+^ ILCs enriched from PBMCs by flow cytometry sorting. Merged plot of blood ILCs from nine samples (N = 3 healthy donors, N = 3 HCC patients at baseline, N = 3 HCC patients after ICI with tremelimumab and durvalumab). Unbiased graph-based clustering reveals 6 clusters. Detailed DEGs of clusters are presented in **Table 1**. **(B–D)** Expression of main ILC marker within UMAP plot. **(B)** Expression of *CD6* and *KLRF*1 to distinguish two ILC populations. **(C)** Expression of *PTGDR2* to identify ILC2 cluster. **(D)** Expression of *KIT* to identify ILC3 cluster. **(E–G)** Same UMAP plot as in Figure 2A), showing cells split by group. Coloring by graphs-based clustering as in **Figure 2A**). **(E)** UMAP plot of cells from healthy donors (N = 3), **(F)** UMAP plot of cells from HCC patients at baseline (N = 3). **(G)** UMAP plot of cells from HCC patients after ICI therapy with tremelimumab and durvalumab (ICI, N = 3). **(H)** Relative frequency of clusters identified by graph-based clustering in **(A)** within all cells of each cohort. **(I)** Volcano plot of DEGs comparing *KLRF1^hig^* ^h^ ILC cluster 5 with all cells. All DEGs passing threshold of FC>2 and p-value<0.05 are plotted. **(J)** Box plots showing expression of *GNLY*, *NKG7* and *IL2RB* in the six ILC clusters identified by scRNA-seq analysis. **(K)** Volcano plot of DEGs comparing *KLRF1^high^* ILC cluster 5 with CD6*^+^* ILC1 cluster 4. All DEGs passing threshold of FC>2 and p-value<0.05 are plotted. **(L)** Box plots showing expression of *CD6*, *CCL5*, and *CXCR6* in the six ILC clusters identified by scRNA-seq analysis.

Unbiased clustering revealed six helper ILC clusters (**Figure 2A**). We identified two ILC1 clusters (clusters 4 and 5), one ILC2 cluster (cluster 1), two ILC3 clusters (clusters 2 and 3), and one ILC3-like, *KIT* expressing cluster (cluster 6) with fewer cells (**Figure 2A** **, DEGs in** **Table S1**). We have previously shown CD6 to be a reliable marker for ILC1s in liver tissue (4). Expression of CD6 was high in cluster 4 (CD6^+^ ILC1), whereas cluster 5 manifested on the opposite side of the CD6*^+^* ILC1 cluster on the UMAP plot. Cluster 5 showed a new and different profile with high expression of the killer-lectin receptor *KLRF1* (**Figure 2B**). *PTGDR2* expression as signature marker of ILC2s was confined to cluster 1 (**Figure 2C**), whereas *KIT* expression, the main marker of ILC3s, was more heterogeneous with cluster 3 showing the highest expression (**Figure 2D**).

Comparative analysis of relative distribution of clusters in scRNA-seq data of healthy donors and HCC patients before and after immunotherapy, revealed that *KLRF1* ^high^ cluster 5 (**Figure 2E** **, purple circle**) was prominent in healthy donors (**Figures 2E, H**) and nearly absent in HCC patients (**Figures 2F, H**). Interestingly, *KLRF1* ^high^ cluster 5 was more prominent after treme/durva treatment (**Figures 2G, H**).

Analysis of differentially expressed genes between helper ILC clusters revealed that cluster 5 showed upregulation of transcripts specific for cytotoxic NK cells including *EOMES and GZMK* as well as high expression of *KLRF1* and *TBX21*, encoding for the transcription factor Tbet, a marker of ILC1s (**Figure 2I**). *KLRF1^high^* ILCs in cluster 5 expressed markers of cytotoxicity including *PRF1, GNL*Y, and *GZMB*, but also showed high NKG7 as well as IL2RB expression, transcripts which are shared with conventional NK cells (**Figure 2J**) (17). Direct comparison of CD6*^+^* ILC1s in cluster 4 and *KLRF1^high^* ILCs in cluster 5 revealed higher expression of ILC3 transcripts such as *CCR7* and *KIT* within *KLRF1^high^* ILCs, indicating a closer connection of this population to ILC3s (**Figure 2K** and **Table S1**). Therefore, cluster 5 (*KLRF1^high^* ILCs) had more of an ILC1-ILC3 intermediate characteristic expressing markers of cytotoxicity. ILCs in cluster 4 in addition to expression of *CD6* expressed higher levels of *CCL5* as well as *CXCR6* as compared to the other clusters (**Figure 2L**).

Interestingly, comparative analysis of scRNA-seq data revealed transcriptomic heterogeneity of ILC3s in PBMC of HCC patients. ILC3s were divided into two main clusters, 2 and 3 (**Figures 2E–G**, **blue circle**), with cluster 3 showing high expression of *KIT* (**Table S1**) thus being referred to as *KIT^high^* ILC3s. ILC3s of cluster 2 were more prominent in the healthy cohort (**Figures 2E, H**) whereas patients with HCC showed a different composition with increased frequency of *KIT^high^* ILC3s (**Figures 2F, H**). *KIT^high^* ILC3s of cluster 3 increased upon treme/durva treatment (**Figures 2G, H**). Direct comparison of the DEG’s from *KIT^high^* ILC3s of cluster 3 with ILC3s of cluster 2 showed increased expression of *NCR2* (transcript for NKp44*), IL32, TGFB1* as well as *RORC*, indicating a more activated state of ILC3s (**Figure S2G**).

### Flow Cytometry and scRNASeq Reveals Numerical and Molecular Heterogeneity of KLRF1^high^ NK-Like ILCs Before and After Immunotherapy

Our data indicate that checkpoint inhibitors significantly alter ILC composition in PBMC of HCC patients. We performed clustering and differential expression analysis of PBMC samples from individual groups of healthy donors (**Figures S2A, B**), HCC patients at baseline (**Figures S2C, D**) and after immunotherapy (**Figures S2E, F**) separately, which confirmed the previously identified major clusters and their distribution (**Figure 2**). Specifically, the KLRF1^high^ ILC population was changed. KLRF1^high^ ILCs were absent in ICI-naïve HCC patients in the individual analyses (**Figure S2C**). Only healthy donors and patients after treatment with tremelimumab plus durvalumab treatment showed a cluster of KLRF1^high^ ILCs (**Figures S2A, E**).

Based on the results from single cell sequencing, we analyzed PBMCs of HCC patients again by flow cytometry to find a corresponding KLRF1^+^ population as shown in cluster 5 (**Figure 2A**). We added NKp80, the protein encoded by *KLRF1*, to our analysis. NKp80 is a marker typically expressed by NK-cells. Although, we excluded classic NK cells from the analysis by CD94 staining (**Figures S1E** in **Supplementary Material**), fluorescence minus one (FMO) validated gating identified a population of CD161^+^CD94^-^CD56^+^NKp80^+^ ILCs in PBMCs (**Figure S3A**) which corresponds to *KLRF1^high^* ILC (**Figure 2A**, cluster 5). On average, 17% of this cell population expressed c-KIT, but did not express CRTH2, indicating a mixed ILC1-ILC3 phenotype with NK-like features, confirmed by the transcriptomic profile of KLRF1^high^ ILC (cluster 5) from scRNA-seq with markers of ILC1 and ILC3 (**Figure S3B**).

**Figure 3:**
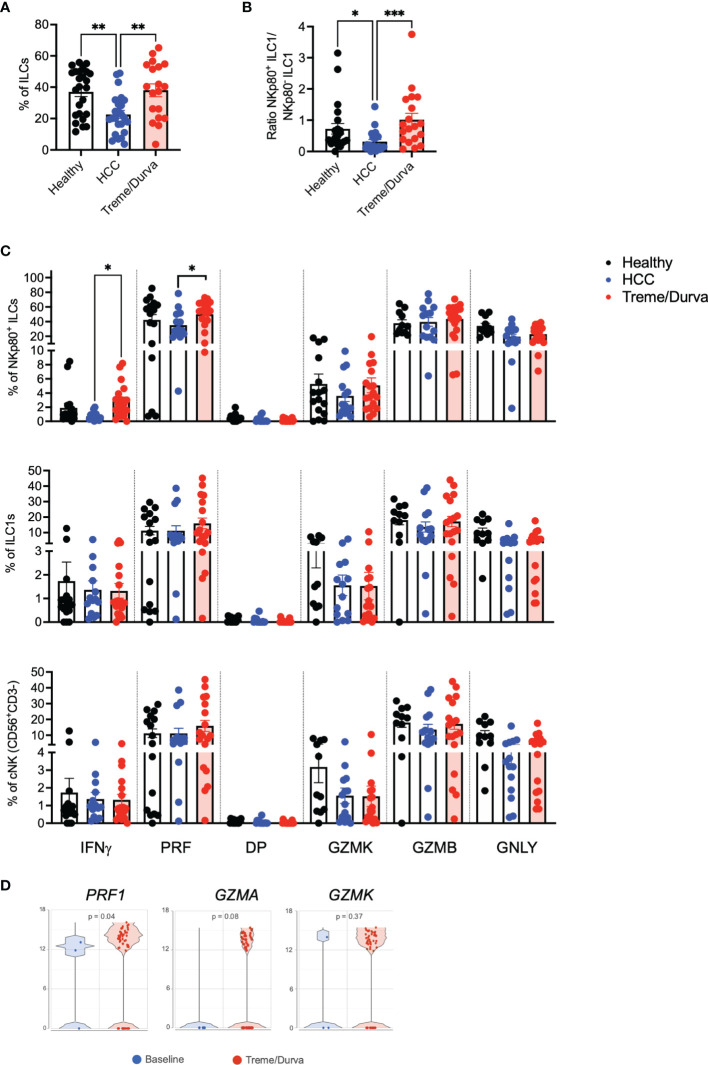
**(A)** Boxplot showing frequencies of NKp80^+^CD56^+^ ILCs of all ILCs in PBMCs of healthy donors (n = 25), patients with HCC (n = 24), or HCC patients after up to four cycles of combined ICI therapy (treme/durva; n = 20)). **(B)** Boxplot showing ratio of NKp80^+^ ILC1s to NKp80^-^ ILC1s in PBMCs of indicated cohorts. **(C)** Frequency of IFNγ, Perforin (PRF), IFNγ and Perforin double positive (DP) and Granzyme K (GZMK), Granzyme B (GZMB) and Granulysin (GNLY) positive cells after stimulation with PMA/Ionomycin for 4h. Cytokine expression comparing NKp80^+^ NK-like cells with ILC1s and conventional NK (cNK) NK cells defined as CD3^-^CD56^+^CD94^+^ cells. **(D)** Volcano plot showing expression of *PRF1*, *GZMA* and *GZMK* in cells of HCC patients at baseline and after ICI (Treme/Durva). Significance: *=p < 0.05, **=p < 0.01, ***=p < 0.001.

Next, we looked for NKp80^+^/KLRF1^high^ ILC population in PBMC of HCC patients before and after ICI therapy. Flow cytometry analysis of PBMCs revealed a significant decrease in the frequency of NKp80^+^/KLRF1^high^ ILCs in peripheral blood of patients with HCC compared to healthy controls (**Figure 3A**, 22.6% vs. 37.03%, p=0.0025). Interestingly, the frequency of NKp80^+^/KLRF1^high^ ILC population was increased to 38.01% after treme/durva treatment similar to levels seen in healthy donors (**Figure 3A**). Furthermore, the ratio of NKp80**^+^** ILC1s compared to NKp80**^-^** ILC1s was increased after double checkpoint inhibitor therapy with treme/durva (**Figure 3B**).

We validated and characterized the NKp80^+/^KLRF1^high^ NK-like population further by performing intracellular cytokine staining and compared them to conventional NK cells (cNKs) defined as CD3^-^CD56^+^CD94^+^ cells. Up to 10% of NKp80^+/^KLRF1^high^ ILCs expressed IFNγ after 4h of stimulation with PMA/Ionomycin. NKp80^+/^KLRF1^high^ ILCs expressed up to 80% perforin. ILC1s and CNKs expressed similar amounts of IFNγ (up to 10%) and some perforin (up to 40%). Compared to NKp80^+/^KLRF1^high^ NK-like cells, cNKs expressed higher amounts of Granzyme K (**Figure 3C**). Furthermore, we compared the expression of cytokines before and after ICI treatment. We saw significant increases in IFNγ (0.7% to 2.89%, p=0.01) and perforin (35.25% to 49.98%, p=0.02) and a trend toward increased GZMK expression (03.6% to 5.1%, p=0.2) in the NKp80^+^ ILC population of patients treated with treme/durva as compared to patients before treatment (**Figure 3C**). Similarly, we saw increases in the expression of perforin, granzyme A and granzyme K in the transcriptomic analyses comparing patients before and after immunotherapy (**Figure 3D**). Natural cytotoxicity receptors (NCRs) NKp30 and NKp44 as well as double positive expression of both NCRs could be found on NKp80^+/^KLRF1^high^ ILCs. ILC1s and NKp80^+^ ILCs mainly expressed NKp44. cNK mainly expressed NKp30 (**Figure S3C**). Additionally, we have analyzed expression of NKp30 and NKp44 on NKp80^+^ ILCs, ILC1s and cNKs before and after ICI treatment (**Figure S3C**). We found significantly reduced expression of NKp30 (9.93% to 2.19%, p=0.010) but increased NKp44 expression (37.93% to 61.08%, p=0.018) in the NKp80^+^ ILC population in HCC patients after ICI treatment. Overall, our data from scRNA-seq and flow cytometry analyses indicates that there is an ILC1-ILC3 intermediate population of NKp80^+/^KLRF1^high^ NK-like cells with a unique expression profile of intermediate expression of c-KIT/*KIT*, cytotoxic potential by expression of perforin, IFNγ and expression of NKp30 and NKp44 as potential functional regulators of this population.

### Trajectory Analysis of ILCs Indicates Plasticity of ILCs in HCC Patients Mediated by Checkpoint Inhibitors

**Figure 4:**
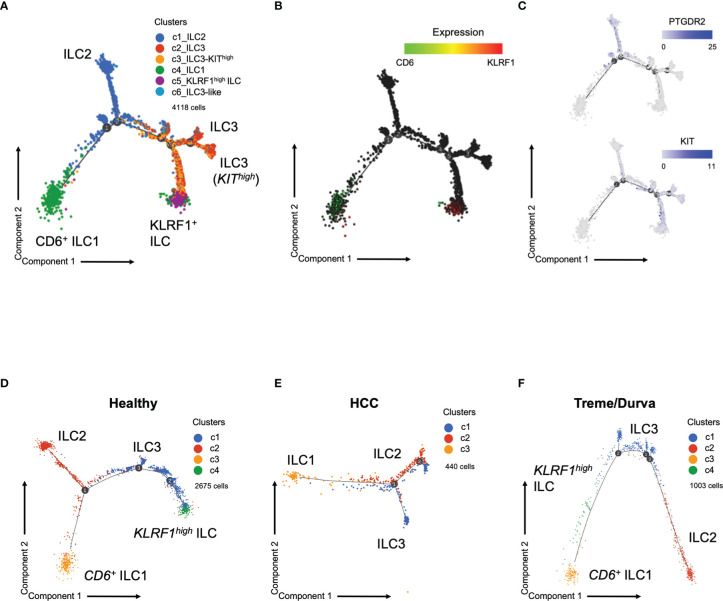
**(A)** Trajectory plot of cells analyzed by scRNA-seq. Same groups and clusters as in **Figure 2A**. **(B)** Expression of ILC marker *CD6* and *KLRF1* in cells on trajectory plot. **(C)** Expression of ILC markers *PTGDR2* and *KIT* in cells on the trajectory plot. D-F) Trajectory plot of cells analyzed by scRNA-seq now showing trajectories split by group. Graph-based clustering and UMAP plot presented in **Figure S2**. **(D)** Trajectory plot of cells from healthy donors (three samples). **(E)** Trajectory plot of cell from HCC patients at baseline (three samples). **(F)** Trajectory plot of HCC patient during immunotherapy with tremelimumab and durvalumab (ICI, three samples).

Next, we performed trajectory analysis to determine the developmental relationship between the six clusters described in **Figure 2A**. Trajectory analysis confirmed that ILC1 cells from PBMC are a highly heterogeneous population with CD6*^+^* ILC1s and *KLRF1^high^* NK-like ILCs at opposite branches, indicating their different transcriptional profiles (**Figures 4A–C**). Comparison of the cohorts showed differences in the branching of trajectory (**Figures 4D–F**). Whereas the *KLRF1^high^* NK-like ILCs and CD6*^+^* ILC1s were at opposite branches in the healthy cohort (**Figure 4D**), patients with HCC did not show the *KLRF^high^* ILC population at all, as we have also shown by flow cytometry (**Figures 3A, B**, **4E**). However, upon ICI therapy the *KLRF1^high^* NK-like ILC population remerged and was placed in between *KIT^hig^* ^h^ ILC3s and ILC1s, indicating a potentially closer relationship and plasticity from either ILC3s or ILC1s, forming the *KLRF1^high^* NK-like ILC cluster under immunotherapy (**Figure 4F**).

#### KLRF1^high^ ILC Signature Is Associated With Better Survival in Large HCC Cohort

**Figure 5:**
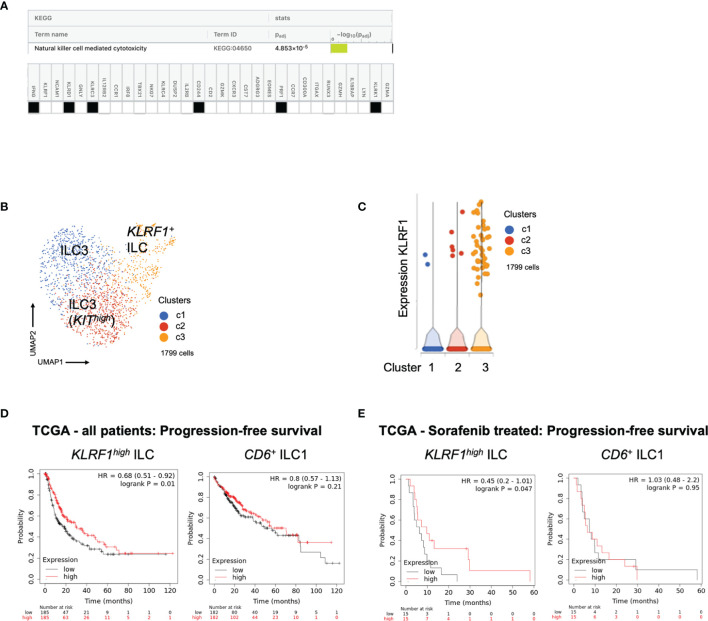
**(A)** Pathway analysis using signature derived from transcripts of *KLRF1^high^* ILCs in PBMCs. KEGG database analysis using gprofiler identifies “Natural killer cell mediated cytotoxicity” as top upregulated pathway. Table shows genes expressed by *KLRF1^high^* ILCs which are involved in the identified pathway. **(B)** UMAP plot of *IL7R^+^KLRB1^+^* ILCs from liver tissue of patients with HCC. Identification of 3 main clusters including two ILC3 clusters and one ILC1 cluster. For detailed DEGs see **Table S1**. **(C)** Violin plot showing expression of *KLRF1* in clusters identified in **Figure 5B**. For detailed DEGs see **Table S1**. **(D)** Analysis of progression-free survival of TCGA liver cancer cohort of patients who have received sorafenib using KaplanMeier-plotter. Survival prediction based on signatures (**Table S1**) derived from transcripts of *KLRF1^high^* ILCs or CD6^+^ ILC1s in PBMCs. Table showing risk stratification of patients. **(E)** Analysis of progression-free survival of TCGA liver cancer cohort of patients who have received sorafenib using Kaplan Meier-plotter. Survival prediction based on signatures derived from transcripts of *KLRF1^high^* ILCs or CD6*^+^* ILC1s in PBMCs. Table showing risk stratification of patients.

Next, we wanted to understand associations to survival in our cohort. Frequency of ILCs over the course of treatment did not show a clear association with response to checkpoint inhibitor therapy, neither in the tremelimumab only cohort (**Figure S4A**) nor in the cohort receiving the combination of tremelimumab and durvalumab (**Figure S4B**). This may be due to the relatively small number of patients included in this study. Interestingly, pathway analysis using upregulated DEGs of the *KLRF1^high^* NK-like ILC population (cluster 5) identified “Natural killer cell mediated cytotoxicity” as the top upregulated pathway in this cell population, indicating its cytotoxic potential (**Figure 5A**). Therefore, we asked whether this signature could be used to predict survival of HCC patients. First, we confirmed that *KLRF1^high^* ILCs can be found not only in PBMCs but also HCC liver tissue. Single cell analysis of liver tissue of HCC patients (n=11) confirmed the presence of *KLRF1^high^* NK-like ILCs in tissue as well (**Figures 5B, C** and **Table S1**). We found similar heterogeneity of ILC3s, with a *KIT^high^* ILC3 population in liver tissue (**Figure 5B** and **Table S1**). Since the correlate of *KLRF1^+^*-ILCs was found in the liver tissue, it provided the rationale for using the signature of *KLRF1^high^* NK-like ILCs for survival analysis in the transcriptomic liver tissue dataset of the larger HCC TCGA cohort. Interestingly, the transcriptomic signature of the *KLRF1^high^* NK-like ILC population correlated significantly with a better progression-free survival (PFS) and recurrence-free survival in the TCGA cohort whereas the signature of CD6*^+^* ILC1s did not predict survival in any cohort (**Figure S4C** **;** **Figure 5D**), indicating a potential role for the *KLRF1^high^* NK-like ILC population in anti-tumor immunity.

Finally, we also performed survival analysis in patients with advanced disease as determined by the former standard treatment with sorafenib in this category of patients. As shown in **Figure 4E**, there was improved PFS in patients with higher expression of the *KLRF1^high^* NK-like ILC signature (**Figure 5E**). Therefore, combined checkpoint inhibitor therapy could be used to increase the *KLRF1^high^* NK-like ILC and improve survival.

## Discussion

Here, we present a detailed profile of ILCs in peripheral blood of HCC patients before and after immunotherapy with ICIs. ILCs in the context of ICI have been described in pancreatic cancer, where ILC2s were able to amplify PD-1 blockade and promote anti-tumor immunity (18). Another study described ILC3s in colorectal cancer, which express MHC-II and communicate with T-cells and are required to induce a strong anti-PD-1 mediated anti-tumor immunity (19). Here, we have identified a heterogeneous ILC1 and ILC3 population including a *KLRF1^high^* NK-like subgroup which shows cytotoxic properties as well as an ILC1-ILC3 intermediate profile as detected both at antigen and transcriptomic levels. The expression of the signature of this population was associated with better progression- and recurrence-free survival in patients of the TCGA liver cancer cohort.

The heterogeneity of group 1 ILCs has been shown to play an important role in the context of cancer. In tumor-bearing mice, cytotoxic NK-cells expressing the specific marker CD49b could be converted into less cytotoxic helper ILC1s expressing CD49a through a double positive, intermediate population. This effect was mediated by tumor derived TGFβ (20).

Recently, a CD56^+^CD16^-^CD127^+^NKp80^+^CD94^+^c-KIT^-^ ILC1-like population has been identified in blood of patients with acute myeloid leukemia with a similar profile to various developmental stages of NK cells. With a signature of CD56^+^CD16^-^CD127^+^NKp80^+^CD94^-^c-KIT^-/+^, the herein described *KLRF1^high^* NK-like population is similar to the previously described CD56^+^ILC-like population but distinct in its CD94 and c-KIT expression. Correlates of the CD56^+^ ILC1-like population could be found in other tissues, but the liver has thus far not been tested (21). Compared to developmental stages of NK cells, the here described population shows a profile of an interim stage between a stage 4b and stage 5 NK cell population, being CD16^-^ and CD94^-^ and partly expressing c-KIT. Specifically, our *KLRF1^high^* NK-like ILC population expressed CD127, the marker for identification of helper ILCs, indicating its ILC1-like state (22, 23). NKp80 has been described to be the critical step between stage 4a and stage 4b development of NK cells and being involved in maturation and development of cytolytic granules (24). Similarly, we have found that the *KLRF1^high^* NK-like ILCs express markers of cytotoxicity which are further increased upon treme/durva treatment. We also identified a correlate of the *KLRF1^high^* NK-like cells in the liver tissue of HCC patients. Since more typical helper ILC1s did not show such strong expression of cytotoxicity transcripts and did not correlate with PFS, the data indicate that the composition of the *KLRF1^high^* NK-like ILC subgroup is associated with anti-tumor immunity.

ILC3s expressed the highest number of immune checkpoints and are the biggest fraction of ILCs, both in blood and liver tissue. ILC3s along with c-KIT^+^ NK cells have been shown to develop from a CD56^+^ ILC precursor but can also transition into each other (25). The trajectory analysis of ILCs in blood identified a close connection between *KLRF1^high^* NK-like ILCs with ILC3s. Tumor development seems to influence composition and plasticity of ILC, since HCC patients showed less *KLRF1^high^* NK-like ILCs and a different trajectory compared to healthy people. Trajectory analysis of ILCs from checkpoint inhibitor treated tumor patients placed *KLRF1^high^* NK-like ILCs in between ILC1s and ILC3s. HCC patients showed specifically an activated state of ILC3s expressing NCR2 transcript and NKp44 surface antigen. This might indicate that these ILC3s can develop into *KLRF1^high^* NK-like ILCs under checkpoint inhibitor therapy and thus promote anti-tumor immunity. We have previously shown that HCC tumor microenvironment modifies ILC composition and induces plasticity amongst the ILC subsets (4).

A more detailed cell fate analysis, which is difficult to address in humans, is needed to prove this hypothesis. Additionally, other immune cells affected by checkpoint inhibitors, specifically T cells and monocytes, might influence ILC heterogeneity through altered cytokine expression. Furthermore, our data indicate that combination therapy changes ILC composition back to similar frequencies in healthy donors. High PD-1 expression on ILC3s and no effect from tremelimumab treatment alone indicates that PD-1 blockade is mainly responsible for this effect. However, more complex interactions cannot be excluded, and it would be rather beneficial to further promote cytotoxic *KLRF1^high^* ILCs, which should be explored in upcoming immunotherapy single agent and combination strategies.

Overall, our data provides evidence that ILCs in the blood undergo plastic transition. Intermediate populations like the here presented NKp80^+^/*KLRF1^high^* ILCs are involved in plasticity and alter the composition of ILC populations. Plasticity seems to be mediated by immunotherapy, although the full mechanism needs to be further explored. The best comparison would be to obtain biopsy materials from liver tissue before and after ICI treatment, however, HCC patients do not get frequent liver biopsies so that only PBMCs are available for sequential analyses.

Here, we show that ILCs and specifically their subgroups need to be included in further analysis of patient cohorts which receive checkpoint inhibitor therapy. Our data suggest that blood ILCs can reflect the influence of checkpoint inhibitors on the tumor microenvironment and thus provide an easy access to study changes under immunotherapy. scRNA-seq can provide a sophisticated method to study even the smallest subgroups in detail, which is required to explore the heterogeneous landscape of ILCs and their plastic and developmental stages. Our analysis helped to identify an NK-like-intermediate ILC population in blood and its transcriptomic signature, which can be used to predict progression- and recurrence-free survival in patients with HCC. This population was increased after checkpoint inhibitor therapy and future studies need to determine if this population is also affected by other immunotherapies and can be used to monitor treatment or can be manipulated to improve efficacy of immunotherapy.

## Data Availability Statement

The original contributions presented in the study are publicly available. This data can be found here: https://www.ncbi.nlm.nih.gov/geo/ under the accession numbers GSE195648 and GSE179795.

## Ethics Statement

The studies involving human participants were reviewed and approved by NCI Institutional Review Board. The patients/participants provided their written informed consent to participate in this study.

## Author Contributions

Project design: BH, TG, and FK. Completion of experiments: BH, BR, VS, and YM. Data analysis and interpretation: BH, BR, VS, YM, WL, AC, JF, CX, AK, TG, and FK. Writing original draft: BH and FK. Review and editing: all authors. All authors contributed to the article and approved the submitted version.

## Funding

TG was supported by the Intramural Research Program of the NIH, NCI (ZIA BC 011344, ZIA BC 011870). AK is supported by the National Institute of Allergy and Infectious Diseases (R01AI132389; R21AI130800). BR was supported by the International Liver Cancer Association (ILCA) Fellowship Award 2021. This work utilized the computational resources of the NIH HPC Biowulf cluster (http://hpc.nih.gov).

## Conflict of Interest

The authors declare that the research was conducted in the absence of any commercial or financial relationships that could be construed as a potential conflict of interest.

## Publisher’s Note

All claims expressed in this article are solely those of the authors and do not necessarily represent those of their affiliated organizations, or those of the publisher, the editors and the reviewers. Any product that may be evaluated in this article, or claim that may be made by its manufacturer, is not guaranteed or endorsed by the publisher.

## Supplementary Material

Supplementary Figure 1
**(A)** Pipeline for analysis of blood samples from healthy donors, patients with hepatitis but no liver cancer, and patients with HCC before and during immunotherapy treatment. PBMCs were isolated and further were stained for ILC marker and analyzed by flow cytometry or enriched for ILCs by flow cytometry sorting and further analyzed by scRNA-seq. Created with BioRender.com. **(B)** Distribution of hepatitis B (HBV) or hepatitis C (HCV) infection within cohorts of hepatitis patients without liver cancer. **(C)** Representative contour plots of flow cytometry data showing gating and sorting strategy for ILCs. **(D)** Distribution of hepatitis B (HBV) or hepatitis C (HCV) infection of patients with HCC enrolled in treatment study with tremelimumab and durvalumab. **(E)** Distribution of hepatitis B (HBV) or hepatitis C (HCV) infection of patients with HCC enrolled in treatment study with tremelimumab. **(F)** Clinical and demographic characteristics of HCC patients analyzed for ILCs in PBMCs enrolled in NCI studies NCT02821754 using combined anti-CTLA-4 (tremelimumab) and anti-PD-L1 (durvalumab) therapy or NCT01853618 using anti-CTLA-4 (tremelimumab therapy). Six patients enrolled in NCT02821754 and all patients of NCT01853618 received some type of interventional radiology (IR) therapy.

Supplementary Figure 2 UMAP plot showing clusters identified by unbiased graph-based clustering of IL7R^+^, KLRB1^+^ ILCs from PBMCs. Table with top upregulated DEGs by cluster. Expression of ILC defining marker *KLRF1* for NK-like ILCs, *CD6* for ILC1s, *PTGDR2* for ILC2s and *KIT* for ILC3s. **(A)** UMAP plot showing clustering and **(B)** top 10 DEGs by cluster of ILCs in PBMCs of healthy donors. **(C)** UMAP plots showing clustering and D) top 10 DEGs by cluster of ILCs in PBMCs of patients with HCC before immunotherapy. **(E)** UMAP plots showing clustering and **(F)** top 10 DEGs by cluster of ILCs in PBMCs of patients with HCC during immunotherapy with checkpoint inhibitors tremelimumab and durvalumab. **(G)** Volcano plot of DEGs comparing ILC3s of cluster 2 with *KIT^high^* ILC3 of cluster 3. All DEGs passing threshold of FC>2 and p-value<0.05 are plotted.

Supplementary Figure 3
**(A)** Gating strategy and fluorescence minus one (FMO) controls to identify CD127^+^ CD161^+^CD94^-^lineage^-^NKp80^+^CD56^+/-^ ILC population. Cells were stained with two different fluorochromes for CD94 as per ILC lin definition to exclude typical NK cells. **(B)** Representative contour plot of flow cytometry data showing expression of NKp80^+^ CD56^+/-^ ILC population and the expression of c-KIT and CRTH2 within this population. **(C)** Frequency of NKp30, NKp44 and NKp30^+^NKp44^+^ double positive cells comparing NKp80^+^ NK-like cells with ILC1s and typical NK cells defined as CD3^-^CD56^+^CD94^+^ cells. Significance: *=p < 0.05

Supplementary Figure 4
**(A)** Frequencies of ILC subgroups over the course of treatment with immunotherapy using tremelimumab, split by response to therapy. C=cycle with C1 representing baseline before therapy. **(B)** Frequencies of ILC subgroups over the course of treatment with immunotherapy using tremelimumab in combination with durvalumab, split by response to therapy. C=cycle with C1 representing baseline before therapy. **(C)** Analysis of recurrence-free survival in TCGA liver cancer cohort of all patients using KaplanMeier-plotter. Survival prediction based on signatures derived from transcripts of *KLRF1^high^* ILCs or CD6*^+^* ILC1s in PBMCs. Table showing risk stratification of patients.

Supplementary Table 1 DEGs of clusters from unbiased graph-based clustering of ILCs in PBMCs.

## Abbreviations

ILC, Innate lymphoid cells; HCC, Hepatocellular carcinoma; ICI, immune checkpoint inhibitor; PD-1, programmed cell death protein 1; PD-L1, programmed cell death 1 ligand 1; CTLA-4, cytotoxic T-lymphocyte-associated protein 4.
