# Supplementary figures and images for: Checkpoint Inhibitors Modulate Plasticity of Innate Lymphoid Cells in Peripheral Blood of Patients With Hepatocellular Carcinoma
Source: Front Immunol. 2022 Jun 27;13:849958. doi: 10.3389/fimmu.2022.849958 (PMC9271772; doi:10.3389/fimmu.2022.849958)

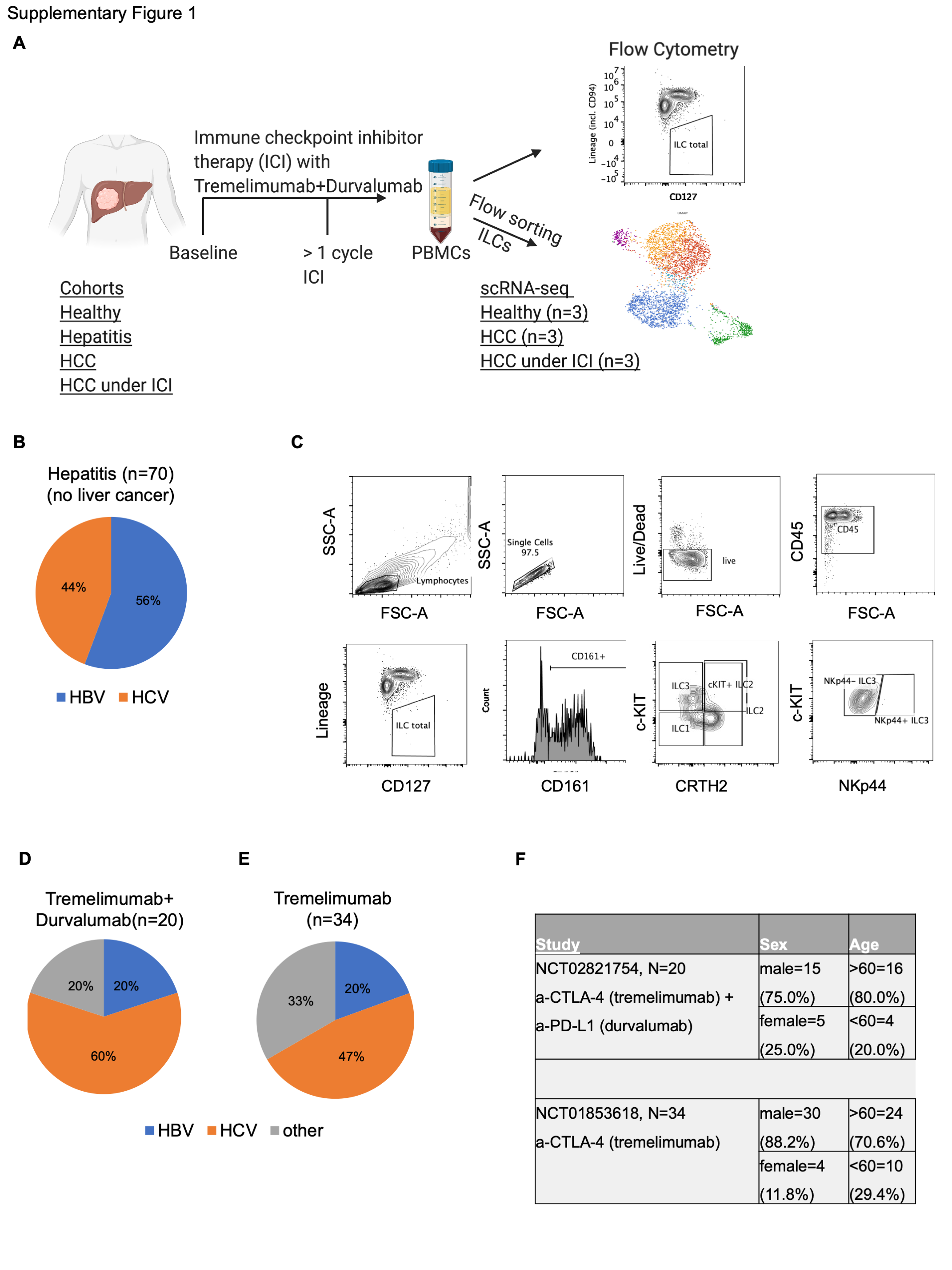

Supplement: Supplementary Figure 1 — (A) Pipeline for analysis of blood samples from healthy donors, patients with hepatitis but no liver cancer, and patients with HCC before and during immunotherapy treatment. PBMCs were isolated and further were stained for ILC marker and analyzed by flow cytometry or enriched for ILCs by flow cytometry sorting and further analyzed by scRNA-seq. Created with BioRender.com. (B) Distribution of hepatitis B (HBV) or hepatitis C (HCV) infection within cohorts of hepatitis patients without liver cancer. (C) Representative contour plots of flow cytometry data showing gating and sorting strategy for ILCs. (D) Distribution of hepatitis B (HBV) or hepatitis C (HCV) infection of patients with HCC enrolled in treatment study with tremelimumab and durvalumab. (E) Distribution of hepatitis B (HBV) or hepatitis C (HCV) infection of patients with HCC enrolled in treatment study with tremelimumab. (F) Clinical and demographic characteristics of HCC patients analyzed for ILCs in PBMCs enrolled in NCI studies NCT02821754 using combined anti-CTLA-4 (tremelimumab) and anti-PD-L1 (durvalumab) therapy or NCT01853618 using anti-CTLA-4 (tremelimumab therapy). Six patients enrolled in NCT02821754 and all patients of NCT01853618 received some type of interventional radiology (IR) therapy. [file Image_1.tiff]

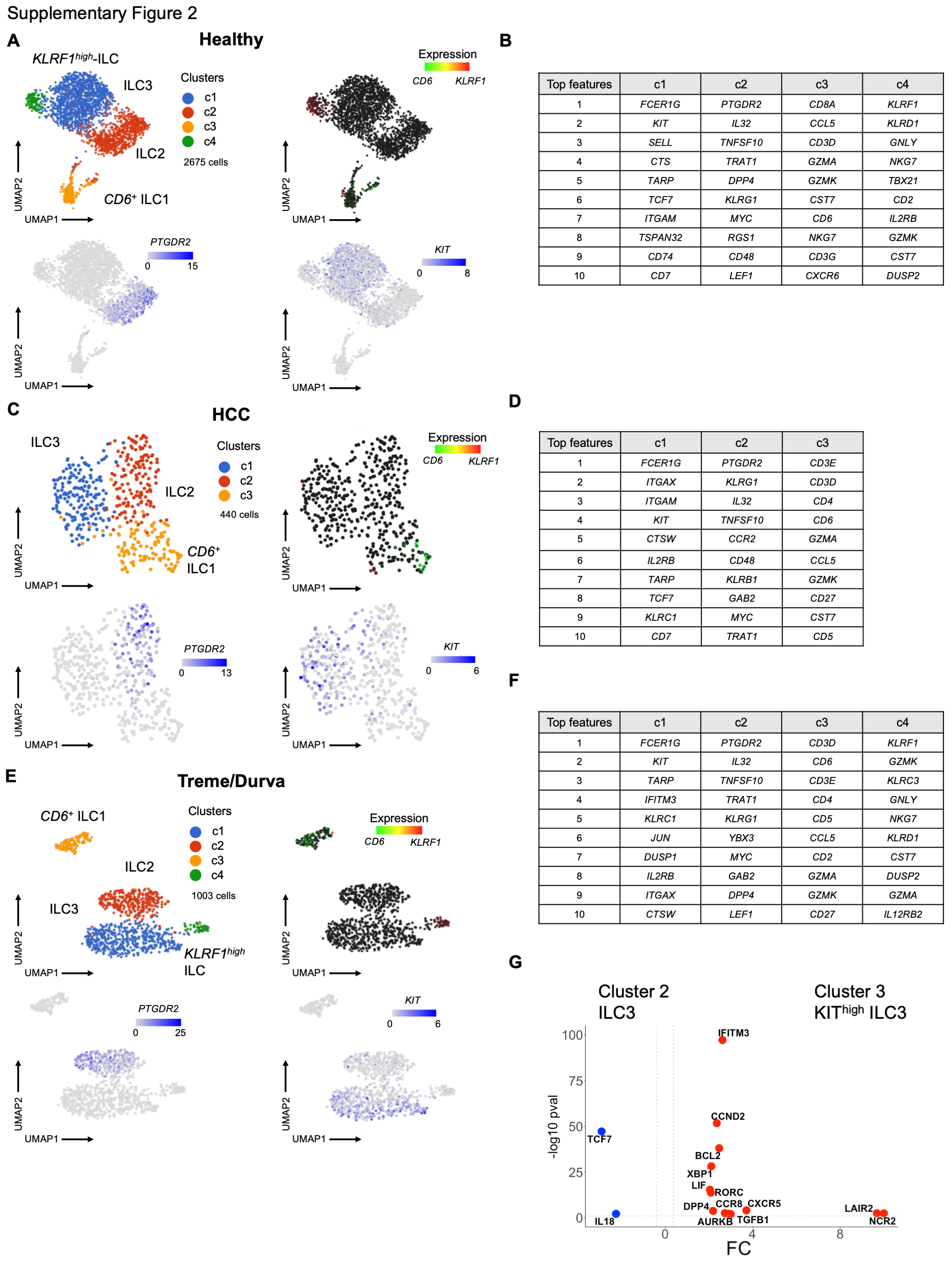

Supplement: Supplementary Figure 2 — UMAP plot showing clusters identified by unbiased graph-based clustering of IL7R+, KLRB1+ ILCs from PBMCs. Table with top upregulated DEGs by cluster. Expression of ILC defining marker KLRF1 for NK-like ILCs, CD6 for ILC1s, PTGDR2 for ILC2s and KIT for ILC3s. (A) UMAP plot showing clustering and (B) top 10 DEGs by cluster of ILCs in PBMCs of healthy donors. (C) UMAP plots showing clustering and D) top 10 DEGs by cluster of ILCs in PBMCs of patients with HCC before immunotherapy. (E) UMAP plots showing clustering and (F) top 10 DEGs by cluster of ILCs in PBMCs of patients with HCC during immunotherapy with checkpoint inhibitors tremelimumab and durvalumab. (G) Volcano plot of DEGs comparing ILC3s of cluster 2 with KIThigh ILC3 of cluster 3. All DEGs passing threshold of FC>2 and p-value<0.05 are plotted. [file Image_2.tiff]

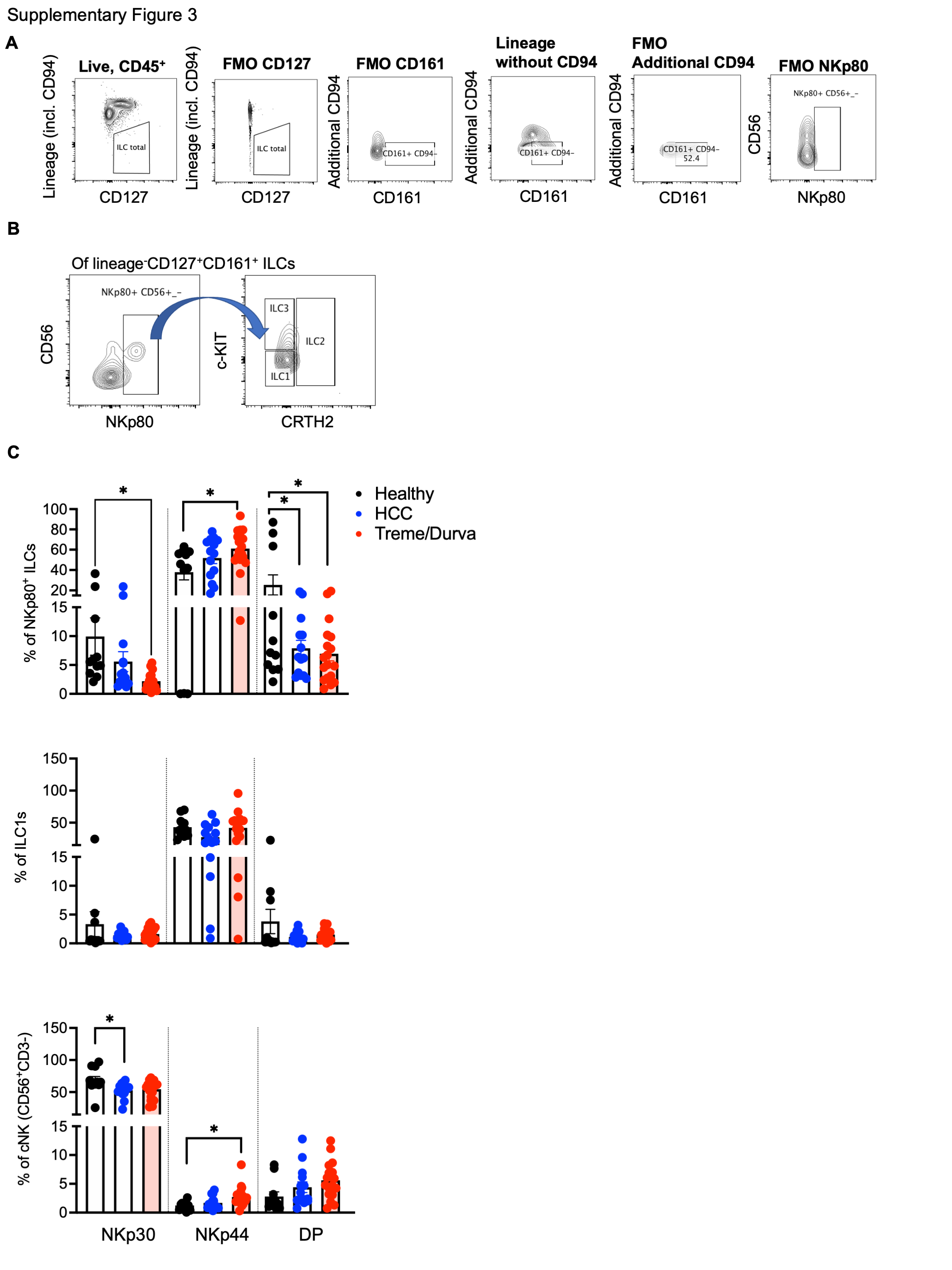

Supplement: Supplementary Figure 3 — (A) Gating strategy and fluorescence minus one (FMO) controls to identify CD127+ CD161+CD94-lineage-NKp80+CD56+/- ILC population. Cells were stained with two different fluorochromes for CD94 as per ILC lin definition to exclude typical NK cells. (B) Representative contour plot of flow cytometry data showing expression of NKp80+ CD56+/- ILC population and the expression of c-KIT and CRTH2 within this population. (C) Frequency of NKp30, NKp44 and NKp30+NKp44+ double positive cells comparing NKp80+ NK-like cells with ILC1s and typical NK cells defined as CD3-CD56+CD94+ cells. Significance: *=p < 0.05 [file Image_3.tiff]

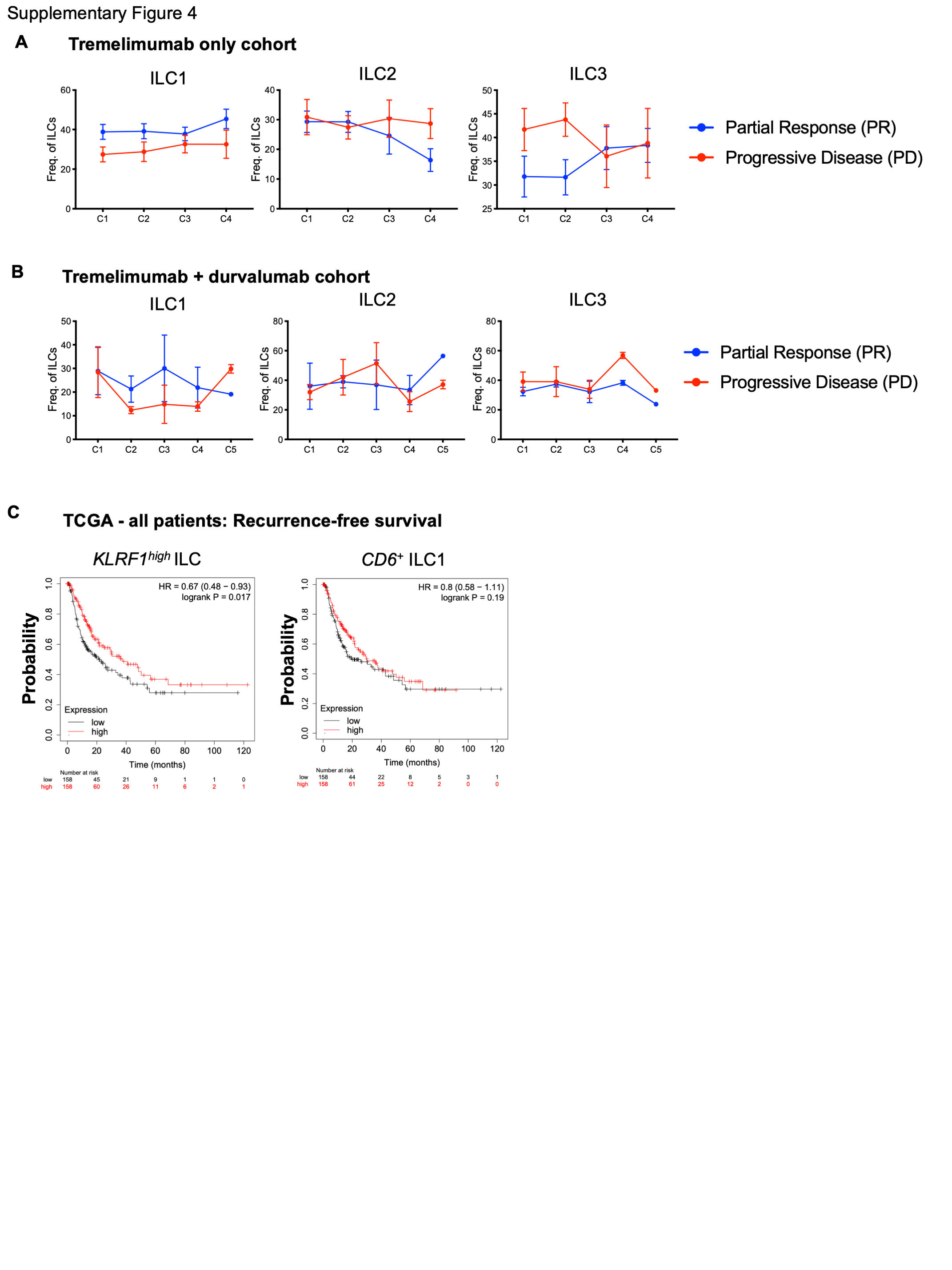

Supplement: Supplementary Figure 4 — (A) Frequencies of ILC subgroups over the course of treatment with immunotherapy using tremelimumab, split by response to therapy. C=cycle with C1 representing baseline before therapy. (B) Frequencies of ILC subgroups over the course of treatment with immunotherapy using tremelimumab in combination with durvalumab, split by response to therapy. C=cycle with C1 representing baseline before therapy. (C) Analysis of recurrence-free survival in TCGA liver cancer cohort of all patients using KaplanMeier-plotter. Survival prediction based on signatures derived from transcripts of KLRF1high ILCs or CD6 + ILC1s in PBMCs. Table showing risk stratification of patients. [file Image_4.tiff]
